# Enhancing Neurovascular Assessment Documentation in Paediatric Supracondylar Fractures: A Closed Loop Audit

**DOI:** 10.7759/cureus.89644

**Published:** 2025-08-08

**Authors:** Ahmed Mohamed, Usman Fuad, Sagaurav Shrestha, Alaa Elasad

**Affiliations:** 1 Trauma and Orthopaedics, Royal Cornwall Hospital, Truro, GBR; 2 General Practice, Zagazig University, Zagazig, EGY

**Keywords:** boast guidelines, closed-loop audit, neurovascular assessment, paediatric fractures, quality improvement, supracondylar fractures

## Abstract

Background and objective

Paediatric supracondylar fractures of the humerus are associated with significant neurovascular complications that can result in devastating long-term consequences if not properly assessed and documented. The British Orthopaedic Association Standards for Trauma and Orthopaedics (BOASts) guidelines emphasise the critical importance of comprehensive neurovascular assessment in these patients. This closed-loop audit aimed to evaluate and improve compliance with BOASts guidelines for preoperative neurovascular documentation in paediatric supracondylar fractures at a District General Hospital in the United Kingdom.

Methods

A retrospective closed-loop audit was conducted over two cycles. The first cycle (January-September 2022) included 24 patients under 15 years with supracondylar fractures. Following implementation of targeted interventions, including staff education and introduction of standardised neurovascular assessment proformas, a second cycle (October 2022-June 2023) was conducted with 34 patients. Data were collected from electronic databases and analysed using Microsoft Excel (Microsoft Corporation, Redmond, Washington, United States).

Results

Compliance with the BOASts guidelines improved significantly from 45.8% (11/24 patients) in the first cycle to 76.5% (26/34 patients) in the second cycle (p = 0.017). Preoperative neurovascular compromise was identified in 12.5% (3/24) of patients in cycle one and 14.7% (5/34) in cycle two, with all cases resolving postoperatively. One patient in each cycle experienced postoperative neurovascular complications, both of which were successfully managed.

Conclusion

Implementation of targeted educational interventions and standardised documentation tools significantly improved compliance with the BOASts guidelines for neurovascular assessment in paediatric supracondylar fractures. This audit demonstrates the effectiveness of systematic quality improvement initiatives in enhancing patient safety and clinical documentation standards.

## Introduction

Paediatric supracondylar fractures of the humerus represent the most common elbow injury in children, accounting for approximately 50-60% of all paediatric elbow fractures [1]. This fracture classification has been described in detail by Gartland, who classified it into three main categories [2]. Type 1 is the most stable and non-displaced, type 2 is displaced with intact posterior cortex, and type 3 is displaced without any cortical contact. The anatomical proximity of the median, anterior interosseous, radial, and ulnar nerves, along with the brachial artery, to the supracondylar region creates a high risk of neurovascular compromise, with reported rates of primary nerve injury ranging from 11-15% and vascular injuries occurring in 3.2-14.3% of cases [3,4]. These complications can result in devastating long-term consequences if not promptly recognised and appropriately managed. Therefore, urgent reduction and stabilisation in cases of vascular compromise is mandatory.

The British Orthopaedic Association Standards for Trauma and Orthopaedics (BOASts) guidelines include strict and clear rules for managing paediatric supracondylar fractures [5]. These should include a comprehensive neurovascular assessment and documentation of individual nerve function of the radial, ulnar, median, and anterior interosseous nerves, as well as assessing radial pulse and capillary refill time before and after the operation. The guidelines also include recommendations for timing of surgery, method of fixation, and type and size of metalwork that should be used. Despite these clear recommendations, existing literature demonstrates significant deficiencies in neurovascular assessment and documentation practices across emergency departments and orthopaedic units [6,7].

This closed-loop audit was conducted to evaluate current compliance with the BOASts guidelines for preoperative neurovascular documentation in paediatric supracondylar fractures at our institution and to implement targeted interventions to improve documentation quality and patient safety outcomes.

## Materials and methods

This closed-loop audit was conducted in the Trauma and Orthopaedics Department at Royal Cornwall Hospital, a District General Hospital in Truro, United Kingdom. The project was approved by the hospital's clinical audit department and supervised by a trauma and orthopaedic consultant. As a quality improvement audit using routinely collected clinical data, formal ethical approval was not required.

Inclusion criteria comprised skeletally immature patients (aged under 15 years) who sustained supracondylar fractures of the humerus requiring surgical management. Exclusion criteria included Gartland 1 classification fractures, as medical records could not be reliably traced, patients aged 15 years or older, and incomplete medical records preventing adequate data extraction for neurovascular assessment evaluation.

Two audit cycles were undertaken. The first cycle was conducted retrospectively from January to September 2022, and the second cycle was conducted retrospectively from October 2022 to June 2023. Data were collected from the hospital's electronic medical records (Bluespier (Clanwilliam, Dublin, Ireland) and eNotes® (eMed Solutions LLC, Miami, Florida, United States)) by reviewing preoperative documentation, operative notes, and follow-up records. Variables collected included patient demographics (age and sex), fracture laterality, fracture pattern classified according to Gartland classification, documented neurovascular assessment components, presence of preoperative neurovascular compromise, surgical approach utilised, postoperative neurovascular status, and clinical outcomes, including complications. The documented preoperative neurovascular assessments were evaluated against the standards set by the BOASts guidelines for supracondylar fractures in children.

Findings from the first audit cycle were presented at local audit meetings and departmental clinical governance meetings. During these meetings, the importance of following the BOASts guidelines was highlighted to ensure clinical accuracy and legal protection for the entire team. Additionally, several strategies were discussed with the audience to improve adherence to the BOASts guidelines. This included developing a sustainable and department-wide educational approach to raise awareness among all team members across the emergency department and orthopaedic team. Agreed-upon interventions included sending comprehensive departmental emails emphasising the clinical importance of thorough neurovascular assessment, including the audit results in the departmental induction for new resident doctors, implementing standardised five-parameter assessment protocols, and distributing neurovascular examination proformas throughout relevant clinical areas.

Following the implementation of these measures, a second audit cycle was conducted. Data were collected and analysed using the same parameters and methodology as in the initial cycle. The results demonstrated marked improvement in clinical practice, highlighting the effectiveness of targeted education and standardised documentation tools as sustainable, low-resource, and impactful methods for enhancing compliance with national standards.

Data were analysed using Microsoft Excel 2021 (Microsoft Corporation, Redmond, Washington, United States) with appropriate statistical formulas and functions. Descriptive statistics were used to summarise patient characteristics and outcomes using frequencies and percentages, with 95% confidence intervals (CIs) calculated for all proportions to provide measures of precision. Categorical variables were compared between audit cycles using the chi-square test for independence and the two-proportion z-test to ensure robust statistical evaluation. For outcomes with small expected frequencies, Fisher's exact test was considered the more appropriate statistical method. Effect sizes were calculated, including odds ratios (ORs) with 95% CIs, absolute risk reduction, and number needed to treat. Effect sizes for proportion differences were calculated using Cohen's h, where $h = 2\left( \arcsin\left( \sqrt{p_{1}} \right) - \arcsin\left( \sqrt{p_{2}} \right) \right)$. Cohen's h values are interpreted as small (h = 0.2), medium (h = 0.5), and large (h = 0.8) effects, providing standardised measures of the magnitude of change between audit cycles. Confidence intervals for proportions were calculated using the Wilson score interval method, and appropriate corrections for multiple comparisons were applied where applicable. Data visualisations were created using Excel's built-in charting capabilities. Statistical significance was set at p < 0.05.

## Results

First audit cycle results

Twenty-four patients were included in the first audit cycle. The demographic and fracture characteristics are summarised in Table 1

**Table 1:** First audit cycle - patient demographics and fracture characteristics (N=24)

Parameter	Frequency (Percentage)
Sex	
Female	11 (45.8%)
Male	13 (54.2%)
Laterality	
Left	15 (62.5%)
Right	9 (37.5%)
Fracture Classification	
Flexion type	3 (12.5%)
Extension type:	
- Gartland 2	5 (20.8%)
- Gartland 3	16 (66.7%)

Compliance with BOASts guidelines for neurovascular documentation was suboptimal in the first cycle, with only 11 patients (45.8%, 95%CI: 25.9%-65.8%) meeting the required standard. Thirteen patients (54.2%) had incomplete documentation of neurovascular status, representing significant gaps in adherence to established clinical guidelines. The most common missed documentations included capillary refill time, which was not documented in 11 patients, radial pulse assessment, absent in seven patients, anterior interosseous nerve function, which was not documented in four patients, and three patients had only abbreviated "neurovascular intact" (NVI) documentation without detailed component assessment.

Twenty-one patients (87.5%) underwent closed reduction, while three patients (12.5%) required open reduction. Three patients (12.5%) presented with preoperative neurovascular compromise, all of which resolved postoperatively. One patient developed postoperative ulnar nerve palsy requiring surgical exploration, during which a 1.6 mm K-wire was found transfixing the ulnar nerve. Following wire removal, the patient regained full motor function with only slight residual numbness over the little finger.

Based on first cycle findings, comprehensive interventions were implemented, focusing on staff education about BOASts guidelines recommendations regarding this topic. Staff education included sending emails explaining the audit idea and emphasizing the medicolegal importance of thorough neurovascular documentation, including five parameters (radial artery, capillary refill time, sensory and motor function of radial, median, ulnar, and anterior interosseous nerves). Additionally, standardised assessment proformas were availed in the emergency and orthopaedic departments to help new staff to do a full assessment by just ticking some boxes in the proforma and attaching it to the patient notes.

Second audit cycle results

Thirty-four patients were included in the second audit cycle. The demographic and fracture characteristics are summarised in Table 2.

**Table 2:** Second audit cycle - patient demographics and fracture characteristics (N=34)

Parameter	Frequency (Percentage)
Sex	
Female	17 (50.0%)
Male	17 (50.0%)
Laterality	
Left	22 (64.7%)
Right	12 (35.3%)
Fracture Classification	
Flexion type	2 (5.9%)
Extension type:	
- Gartland 2	15 (44.1%)
- Gartland 3	17 (50.0%)

Compliance with BOASts guidelines improved significantly to 76.5% (26/34 patients, 95% CI: 62.2%-90.7%), representing a 30.6% absolute improvement from the first cycle (p = 0.017). Ten patients (23.5%) continued to have incomplete documentation. The standardised neurovascular documentation proforma was utilised in 17 patients (50.0%) during the second cycle. Detailed analysis of these ten cases revealed that three patients had complete documentation by the orthopaedic team, but radial artery assessment was not documented by emergency department staff prior to cast application. Four patients had only "NVI" (neurovascular intact) documented without detailed assessment, and three patients had partial documentation, missing specific nerve function assessments.

Thirty-three patients (97.1%) underwent closed reduction, while one patient (2.9%) required open reduction. Five patients (14.7%) presented with preoperative neurovascular compromise, all of which resolved postoperatively. One patient developed transient motor neuropathy affecting all nerves supplying the hand, which resolved completely and was attributed to postoperative swelling and pain.

**Figure 1:**
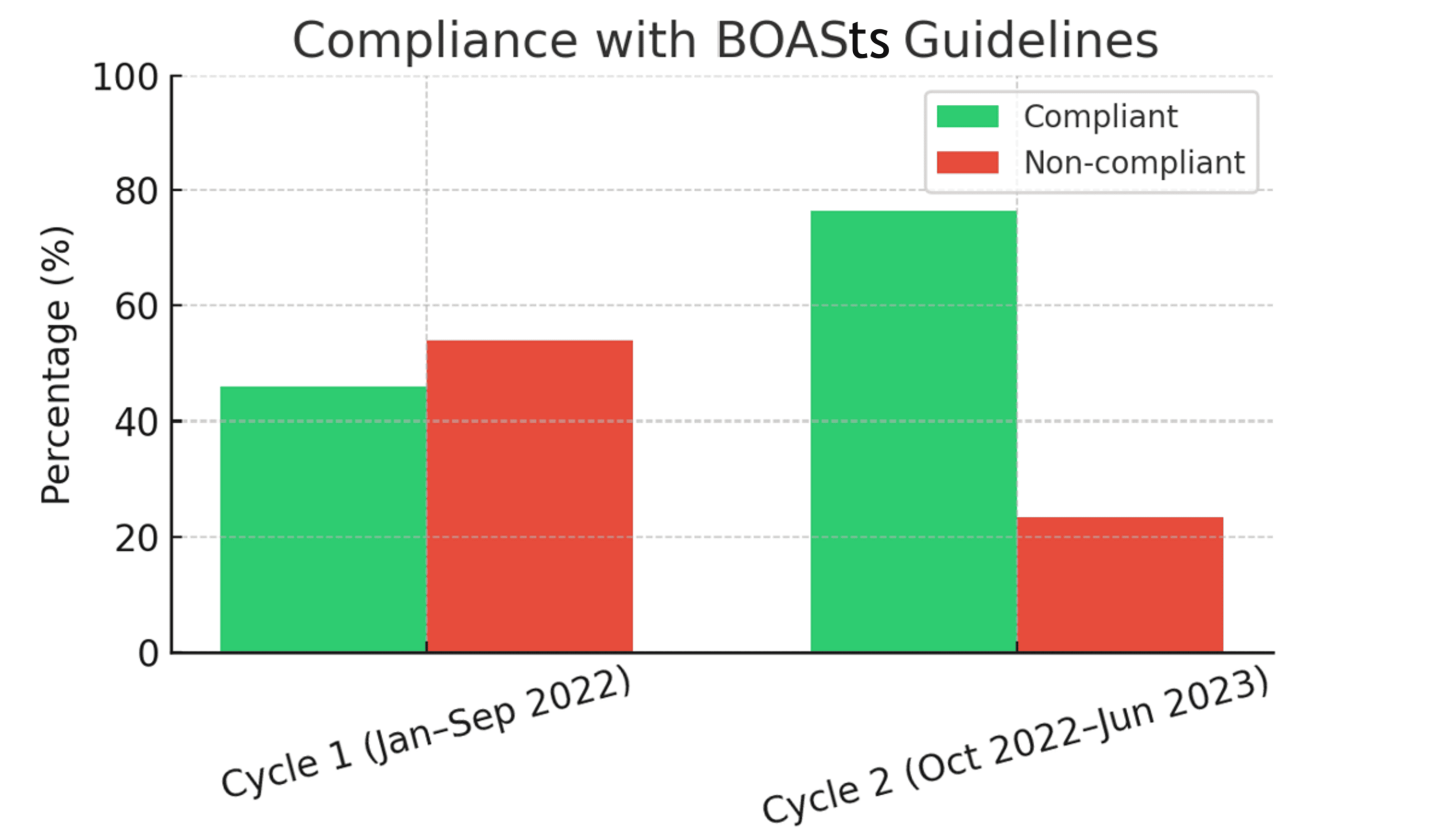
Percentage of patients meeting BOASts documentation standards (compliant vs non-compliant) BOASts: British Orthopaedic Association Standards for Trauma and Orthopaedics

Comparative analysis between audit cycles

The improvement in compliance with BOASts guidelines was substantial and statistically significant. Compliance improved from 45.8% (11/24 patients, 95% CI: 25.9%-65.8%) in the first cycle to 76.5% (26/34 patients, 95% CI: 62.2%-90.7%) in the second cycle. This represents an absolute improvement of 30.6% (95% CI: 6.1%-55.1%) and a relative improvement of 66.8%. The improvement was statistically significant using both chi-square analysis (χ² = 5.717, df = 1, p = 0.017) and two-proportion z-test (z = 2.391, p = 0.017). The number needed to treat was 3.3, indicating that for every 3.3 patients assessed using the improved protocol, one additional patient would have complete neurovascular documentation compared to the previous standard of care.

**Table 3:** Primary and secondary outcomes comparison between audit cycles Statistically significant (p < 0.05) BOASts: British Orthopaedic Association Standards for Trauma and Orthopaedics

Parameter	First Cycle (n=24)	Second Cycle (n=34)	Absolute Difference (95% CI)	Statistical Test	P-value
Primary Outcome					
Compliance with BOASts guidelines	11 (45.8%)	26 (76.5%)	+30.6% (6.1%, 55.1%)	χ² = 5.717	0.017*
Secondary Outcomes					
Preoperative neurovascular compromise	3 (12.5%)	5 (14.7%)	+2.2% (-11.8%, 16.2%)	Fisher's exact	0.73
Open reduction required	3 (12.5%)	1 (2.9%)	-9.6% (-22.4%, 3.2%)	Fisher's exact	0.30
Postoperative complications	1 (4.2%)	1 (2.9%)	-1.3% (-11.8%, 9.2%)	Fisher's exact	1.00

**Table 4:** Effect size measures for primary outcome *this indicates that for every 3.3 patients assessed using the improved protocol, one additional patient achieved complete neurovascular documentation compared to standard practice.

Measure	Value	95% Confidence Interval
Odds Ratio for compliance	3.85	(1.24, 11.92)
Number Needed to Treat	3.3 patients*	-
Cohen's h (effect size)	0.635	-
Absolute Risk Reduction	30.6%	(6.1%, 55.1%)
Relative Risk Improvement	66.8%	-

The improvement in compliance represents a statistically significant enhancement in documentation quality (χ² = 5.717, df = 1, p = 0.017), demonstrating the effectiveness of the implemented interventions. The odds of achieving full compliance were 3.85 times higher in the second cycle compared to the first cycle (95% CI: 1.24-11.92).

## Discussion

This closed-loop audit highlighted two main findings. First, it assessed our departmental adherence to BOASts guidelines for paediatric supracondylar fractures of the humerus. Second, it demonstrated the importance of applying simple, sustainable, and low-cost educational methods in aligning clinical practice with national standards.

Statistical and clinical significance

The 30.6% absolute improvement in compliance with BOASts guidelines represents both statistical significance (p = 0.017) and clinical significance. The effect size (Cohen's h = 0.635) indicates medium to large practical significance of the intervention. The number needed to treat of 3.3 demonstrates that the intervention has a meaningful clinical impact, as fewer than four patients need to be exposed to the improved protocol to achieve one additional case of complete neurovascular documentation.

The confidence interval for the improvement (6.1-55.1%) indicates that even the most conservative estimate suggests a clinically meaningful benefit. The odds ratio of 3.85 (95%CI: 1.24-11.92) demonstrates that patients in the second cycle had nearly four times the odds of receiving a complete neurovascular assessment compared to the first cycle.

The improvement in neurovascular documentation compliance has several important implications for patient care. Comprehensive baseline neurovascular assessment and documentation serve as the reference point that healthcare workers can use for comparison in any future neurovascular assessments during the patient's treatment journey, enabling early recognition of complications [8]. This is particularly important as neurovascular compromise can develop suddenly and may be difficult to detect without any pre-documented reference.

Challenges in paediatric neurovascular assessment

Assessing neurovascular status in paediatric patients presents unique challenges that require specific approaches and awareness from healthcare professionals [9]. Children may be unable to articulate neurological symptoms effectively due to developmental factors, pain, anxiety, and the unfamiliar hospital environment [10]. The trauma itself and its psychological effects can interfere with the child's ability to participate in neurological testing, often resulting in incomplete or unreliable examination findings.

Healthcare providers must develop sophisticated skills in recognising non-verbal indicators of neurological compromise and utilise age-appropriate assessment techniques. The "Rock, Paper, Scissors, OK" method has been shown to improve both patient cooperation and clinical accuracy by transforming a potentially anxiety-provoking examination into an engaging, game-like activity [11].

Clinical outcomes and complication management

The incidence of preoperative neurovascular compromise in our study (12.5% in cycle one, 14.7% in cycle two) aligns with published literature reporting rates between 11-15% for nerve injuries and 3.2-14.3% for vascular injuries [3,4]. Importantly, all preoperative neurovascular deficits resolved postoperatively in both audit cycles, which is consistent with the generally favourable prognosis for nerve injuries in paediatric patients due to enhanced regenerative capacity [12].

The postoperative complications encountered in both cycles highlight the importance of maintaining vigilance during the perioperative period. The ulnar nerve entrapment by K-wire in cycle one demonstrates a recognised iatrogenic complication that requires prompt recognition and surgical correction [13,14]. Several studies have discussed ulnar nerve palsy after cross-wiring for supracondylar fractures. However, the recovery rate from this injury is approximately 90%, and early exploration was not recommended by these studies as the damage has already occurred; therefore, expectant management was advised [15-18].

Intervention effectiveness

The interventions implemented between audit cycles demonstrated high effectiveness in improving documentation compliance. Despite the high importance of BOASts guidelines, they were designed and are followed mainly in healthcare practice in the United Kingdom. The NHS experiences a large number of international medical graduates every year who may not be aware of recent guidelines that need to be followed [19]. Regular auditing and demonstrating results before and after implementation of changes is crucial in bringing attention to guidelines such as BOASts among new staff members. Therefore, departmental education, including guidelines teaching in staff induction, email reminders, and provision of standardised proformas that serve as cognitive aids for busy clinicians, is crucial.

Implications for practice

The results of this audit have several important implications for clinical practice. The importance of ongoing monitoring and re-audit is emphasised by the sustained improvement observed in the second cycle. Regular audit cycles should be considered standard practice to maintain high documentation standards and ensure continuous quality improvement. Finally, this audit demonstrates the importance of the Plan-Do-Study-Act (PDSA) model in improving clinical practice by identifying a gap, introducing a targeted intervention, and reassessing results [20]. This demonstrates the importance of proactivity in discussing departmental gaps and finding solutions to address them.

Limitations

This audit has several limitations that should be acknowledged. The retrospective nature of data collection limits the ability to assess the quality of clinical examination beyond what was documented. Some neurovascular assessments may have been performed but not adequately recorded, leading to potential underestimation of actual compliance rates. The relatively small sample sizes in both cycles may limit the generalisability of findings, although the consistency of results with published literature supports the validity of our observations. The single-centre design may also limit external validity, as practice patterns and resources vary between institutions.

Remaining challenges and future directions

Despite significant improvement, 23.5% of patients in the second cycle still had incomplete neurovascular documentation. Analysis of these cases revealed specific areas for further improvement, including enhanced communication between the emergency department and orthopaedic team regarding assessment timing and responsibilities. The data collection in the second cycle showed that three patients had a full neurovascular assessment by the orthopaedic team except for radial artery documentation, as patients were in above elbow back slab when they were seen, and radial artery assessment was not documented by the emergency department prior to casting. This is potentially dangerous as a pink pulseless hand can be easily missed, which is an indication for urgent reduction and surgical fixation [4,21].

The clinical implications of poor documentation affect both patients and healthcare providers. Inadequate baseline documentation delays recognition and management of developing complications. Furthermore, poor documentation makes differentiating between traumatic and iatrogenic nerve injuries very challenging, which is crucial given that iatrogenic neurapraxias account for approximately 2-3% of nerve injuries in paediatric supracondylar fractures [22]. This subsequently affects decisions regarding both immediate management and long-term prognosis, which puts healthcare workers in difficult situations with patients and families.

The economic impact of missed neurovascular complications represents a burden on the National Health Service. This includes prolonged hospital stays, additional surgical procedures, and long-term rehabilitation that consumes healthcare system resources. This economic impact is avoidable if comprehensive initial assessment and documentation are performed [23,24].

## Conclusions

This closed-loop audit demonstrates that targeted interventions can significantly improve compliance with BOASts guidelines for neurovascular documentation in paediatric supracondylar fractures. The 30.6% improvement in compliance achieved through staff education and the availability of documentation proformas represents a meaningful enhancement in patient safety and clinical standards. The findings support the effectiveness of systematic quality improvement initiatives in healthcare and highlight the importance of regular audit cycles to maintain high standards. While significant progress was achieved, continued efforts are needed to address remaining documentation gaps and ensure optimal patient care.

Future work should focus on developing more assessment tools, including mobile applications or electronic proformas, improving interdisciplinary communication, and exploring the potential for electronic documentation systems to further enhance compliance and patient safety outcomes. The goal remains the prevention of neurovascular complications and optimisation of functional outcomes for children with supracondylar fractures.
